# Analysis of intrahospital and global dissemination and resistome dynamics of NDM-1-producing ST773 *Pseudomonas aeruginosa* high-risk clone

**DOI:** 10.1093/jacamr/dlaf063

**Published:** 2025-04-22

**Authors:** Cristina Pitart, Gabriel Taltavull, Carla López-Causapé, Andrea Pulgarín, Sergi De Gea, Mireia Aguilar, Xavier Mulet, Gabriel Cabot, Jordi Vila, Ignasi Roca, Mateu Espasa, Climent Casals-Pascual, Antonio Oliver

**Affiliations:** Department of Clinical Microbiology, Biomedical Diagnostic Center, Hospital Clinic, Barcelona, Spain; Barcelona Institute for Global Health (ISGlobal), Barcelona, Spain; Department of Basic Clinical Practice, School of Medicine, University of Barcelona, Barcelona, Spain; Servicio de Microbiología, Hospital Universitario Son Espases, Instituto de Investigación Sanitaria Illes Balears (IdISBa), Palma de Mallorca, Spain; CIBER de Enfermedades Infecciosas (CIBERINFEC), Instituto Salud Carlos III, Madrid, Spain; Servicio de Microbiología, Hospital Universitario Son Espases, Instituto de Investigación Sanitaria Illes Balears (IdISBa), Palma de Mallorca, Spain; CIBER de Enfermedades Infecciosas (CIBERINFEC), Instituto Salud Carlos III, Madrid, Spain; Department of Clinical Microbiology, Biomedical Diagnostic Center, Hospital Clinic, Barcelona, Spain; Department of Clinical Microbiology, Biomedical Diagnostic Center, Hospital Clinic, Barcelona, Spain; Department of Clinical Microbiology, Biomedical Diagnostic Center, Hospital Clinic, Barcelona, Spain; Servicio de Microbiología, Hospital Universitario Son Espases, Instituto de Investigación Sanitaria Illes Balears (IdISBa), Palma de Mallorca, Spain; CIBER de Enfermedades Infecciosas (CIBERINFEC), Instituto Salud Carlos III, Madrid, Spain; Servicio de Microbiología, Hospital Universitario Son Espases, Instituto de Investigación Sanitaria Illes Balears (IdISBa), Palma de Mallorca, Spain; CIBER de Enfermedades Infecciosas (CIBERINFEC), Instituto Salud Carlos III, Madrid, Spain; Department of Clinical Microbiology, Biomedical Diagnostic Center, Hospital Clinic, Barcelona, Spain; Barcelona Institute for Global Health (ISGlobal), Barcelona, Spain; Department of Basic Clinical Practice, School of Medicine, University of Barcelona, Barcelona, Spain; CIBER de Enfermedades Infecciosas (CIBERINFEC), Instituto Salud Carlos III, Madrid, Spain; Department of Clinical Microbiology, Biomedical Diagnostic Center, Hospital Clinic, Barcelona, Spain; Barcelona Institute for Global Health (ISGlobal), Barcelona, Spain; Department of Basic Clinical Practice, School of Medicine, University of Barcelona, Barcelona, Spain; CIBER de Enfermedades Infecciosas (CIBERINFEC), Instituto Salud Carlos III, Madrid, Spain; Department of Clinical Microbiology, Biomedical Diagnostic Center, Hospital Clinic, Barcelona, Spain; Barcelona Institute for Global Health (ISGlobal), Barcelona, Spain; Department of Basic Clinical Practice, School of Medicine, University of Barcelona, Barcelona, Spain; Department of Clinical Microbiology, Biomedical Diagnostic Center, Hospital Clinic, Barcelona, Spain; Barcelona Institute for Global Health (ISGlobal), Barcelona, Spain; Department of Basic Clinical Practice, School of Medicine, University of Barcelona, Barcelona, Spain; CIBER de Enfermedades Infecciosas (CIBERINFEC), Instituto Salud Carlos III, Madrid, Spain; Servicio de Microbiología, Hospital Universitario Son Espases, Instituto de Investigación Sanitaria Illes Balears (IdISBa), Palma de Mallorca, Spain; CIBER de Enfermedades Infecciosas (CIBERINFEC), Instituto Salud Carlos III, Madrid, Spain

## Abstract

**Objectives:**

To analyse the intrahospital and global dissemination and resistome dynamics of the concerning NDM-1 MBL-producing ST773 *P. aeruginosa* high-risk clone.

**Methods:**

A total of 17 NDM-1-producing *P. aeruginosa* isolates recovered in 2022–24 from 10 patients at Hospital Clinic of Barcelona (HCB), Spain, were studied through susceptibility testing and WGS. Expression of resistance genes was analysed through quantitative (real-time) RT–PCR. Forty ST773 genomes from isolates recovered worldwide were also incorporated in the phylogenetic and resistome analysis.

**Results:**

All HCB NDM-1-producing isolates were assigned to ST773 except one (ST357 additionally producing VEB-9 and linked epidemiologically to India). The index ST773 case was a 41-year-old woman admitted to the oncology ward in February 2022 after breast cancer surgery in Ukraine. These isolates were closely related and the *bla*_NDM-1_ gene was located in the same 117 kb integrative conjugative element. All ST773-NDM-1 producers from HCB and the 40 worldwide isolates shared the same acquired resistance determinants [*aadA11-*like, *rmtB4*, *qnrVC1* and *tet*(G)], as well as some of the antibiotic resistance mutations (*mexZ*, *mexT*, *gyrA* and *parC*). Other specific mutations such as an *oprD* deletion were shared only with isolates from Ukrainian patients transferred to Madrid or the Netherlands. Lastly, HCB isolates evolved further resistome mutations during intrahospital dissemination, including regulators of AmpC (*mpl*) and MexAB-OprM (*nalD*), linked to the acquisition of aztreonam/avibactam resistance, and thus remaining only susceptible to cefiderocol and colistin.

**Conclusions:**

This work evidences the transborder spread and intrahospital dissemination and evolution of the emerging ST773-NDM-1 *P. aeruginosa* high-risk clone.

## Introduction

The global spread of antibiotic-resistant bacteria poses a significant challenge to public health, with *Pseudomonas aeruginosa* representing a critical threat due to its intrinsic antibiotic resistance and its outstanding ability to develop further resistance by the acquisition and selection of chromosomic mutations and/or the acquisition of additional resistance genes.^1^

A nationwide genomic epidemiological study recently conducted in Spain showed a generalized decrease in *P. aeruginosa* resistance in the last 5 years but an increase in the proportion of XDR strains producing carbapenemases in association with the spread of the high-risk clone ST235.^2^ Likewise, other studies have also suggested that the production of acquired carbapenemases appears to be rising in frequency worldwide.^3^ Among these enzymes, NDM is particularly concerning as it confers resistance to a wide range of β-lactam antibiotics, including carbapenems and newer β-lactam/β-lactamase inhibitor combinations, often considered last-resort treatments for MDR infections.^4^

The emergence of NDM-1-producing *P. aeruginosa* strains, especially in high-risk clones like ST773, exacerbates this threat, as these clones are highly transmissible within healthcare settings and across borders. NDM-1-producing ST773 isolates have already been detected in various countries worldwide, reflecting its potential for widespread dissemination.^5–19^ Understanding both the local and global dissemination patterns, as well as the resistome dynamics of NDM-1-producing ST773 *P. aeruginosa*, is crucial for developing effective infection control strategies and to mitigate the impact of these emerging antibiotic-resistant pathogens.

In this study, we investigated the introduction, dissemination and persistence of an NDM-1-producing *P. aeruginosa* ST773 clone within a tertiary Spanish hospital over a 2 year period. By combining phenotypic and whole-genome approaches we aimed to provide insights into the genetic relatedness and evolution of this high-risk clone worldwide in order to enhance the understanding of the mechanisms driving the global spread of the NDM-1-producing ST773 *P. aeruginosa* high-risk clone.

## Materials and methods

### *P. aeruginosa* strains and antibiotic susceptibility testing

From April 2022 to February 2024, NDM-producing *P. aeruginosa* isolates were obtained from 10 different patients admitted to the Hospital Clinic of Barcelona (HCB), an 850-bed tertiary hospital, in both clinical samples and surveillance rectal swabs.

MICs of piperacillin/tazobactam, ceftazidime, cefepime, ceftolozane/tazobactam, ceftazidime/avibactam, imipenem, meropenem, ciprofloxacin, tobramycin, amikacin and colistin were determined by broth microdilution using Sensititre^™^ panels (plate code: FRCNRP2, Thermo Fisher Diagnostics, S.LU.). To determine the MICs of aztreonam, aztreonam/avibactam and cefiderocol, an in-house broth microdilution was performed according to ISO-EUCAST guidelines (http://www.eucast.org) using CAMHB for aztreonam and aztreonam/avibactam, and ID-CAMHB for cefiderocol. EUCAST clinical breakpoints (v14) were used for interpretation of SIR categories (except for aztreonam/avibactam). The reference strain PAO1 and its knockout mutant PAOΔ*piuC* were included in the susceptibility testing experiments as quality controls.

Since most of the isolates were identified as ST773, an emerging high-risk clone of *P. aeruginosa* reported in various countries, we conducted a thorough search of the PubMed database to compare our isolates with those described globally. The search was based on the following criteria: ST773 [OR] NDM [AND] *P. aeruginosa* and, when publicly available, reads and/or draft genomes were downloaded for further analysis. (ST773-NDM-1 producers: ERR3181579, ERR3181597, ERR3181683, GCA_017292115, GCA_023995335, GCF_003725635, GCF_003954355, GCF_003954525, GCF_009664165, GCF_009791355, GCF_013255565, GCF_018448985, GCF_028595405, GCF_028595445, GCF_028595535, GCF_028595585, GCF_028595705, GCF_028595795, GCF_036321415, GCF_036321435, GCF_036321965, GCF_036321985, GCF_036322045, GCF_036418295, SRR23955892, SRR23955894;^5–10,12,16^ ST773-NDM-1 non-producers: GCF_000790805, GCF_000791735, GCF_000796095, GCF_002411915, GCF_003585175, GCF_003836135, GCF_007559065, GCF_017693465, GCF_017693745, GCF_017693755, GCF_021245785, GCF_021245865, GCF_028595895, GCF_900636975.^9,12,20^

### WGS and resistome analysis

Genomic DNA was obtained with a commercially available extraction kit (High Pure PCR Template Preparation Kit, Roche Diagnostics) and indexed paired-end libraries were prepared with the Illumina DNA Prep Library Preparation Kit (Illumina) and sequenced on an Illumina MiSeq^®^ benchtop sequencer using a MiSeq reagent kit v3 600 cycles (Illumina).

Short reads were *de novo* assembled with SPAdes v3.15.5 (−careful) and the *de novo* assemblies were used to infer the ST by using MLST v2.09 according to PubMLST typing schemes,^21^ to explore the presence of horizontally acquired antimicrobial resistance genes (http://genepi.food.dtu.dk/resfinder) and to study the structural integrity of the OprD porin. In addition, a variant calling analysis was performed to study the *P. aeruginosa* mutational resistome; for this purpose, Snippy software v4.6.0 (https://github.com/tseemann/snippy) was used using the *P. aeruginosa* PAO1 genome (NC_002516.2) as reference. SNPs and short insertions and deletions (InDels) found in the *P. aeruginosa* mutational resistome genes were extracted and polymorphisms were filtered.^22^ The levels of expression, in comparison with the *P. aeruginosa* reference strain PAO1, of the genes encoding the chromosomal β-lactamase AmpC (*ampC*) and the three *P. aeruginosa* efflux pump components [*mexB* (MexAB-OprM), *mexD* (MexCD-OprJ) and *mexY* (MexXY-OprM)], were determined in selected isolates by quantitative (real-time) RT–PCR (RT–qPCR) with an Eco real-time PCR system (Illumina), according to previously described protocols.^23^

### Genomic characterization of the bla_NDM-1_ genetic environments

Representative isolates (HCB22-1-0337, HCB22-1-0461 and HCB23-7-1060) were subjected to Oxford Nanopore long-read sequencing to further investigate the *bla*_NDM-1_ genetic environments. For this, genomic DNA was obtained with the Monarch^®^ Genomic DNA Purification Kit (New England Biolabs) and libraries were prepared as per the manufacturer’s protocol using the SQK-LSK114 Ligation Sequencing Kit v14 and the SQK-NBD114.24 Native Barcoding Kit and loaded onto a MinION flow cell vR10.4.1 and sequenced in a GridION^TM^ device (Oxford Nanopore Technologies).

Complete hybrid genomes were obtained combining short and long reads using the *de novo* hybrid assembler hybracter v0.7.3^24^ and annotated with Bakta v1.9.4.^25^ Finally, the *bla*_NDM-1_ genetic environments were visualized and plotted using Proksee (https://proksee.ca.)^26^

### Phylogenetic analysis and comparison with worldwide isolates of *P. aeruginosa* ST773

To infer the genetic relatedness and possible transmission events among patients, a core SNP-based maximum-likelihood tree was performed using Parsnp v1.2 from the Harvest Suite package, forcing the inclusion (-c) of all genomes. In addition, a core-genome MLST (cgMLST) was performed using the open-source algorithm chewBBACCA;^27^ allele matrix distances were obtained and a minimum spanning tree was constructed with GrapeTree.^28^

## Results and discussion

### Antibiotic susceptibility profiles and resistome characterization of NDM-1-producing *P. aeruginosa* isolates detected at HCB

A total of 17 NDM-1-producing *P. aeruginosa* isolates obtained from 10 different patients were studied (Table 1). All isolates were assigned to the emerging high-risk clone ST773, with the single exception of isolate HCB23-6-1048 (Patient 6), which was assigned to ST357.

**Table 1.** Antibiotic resistance profiles and resistome of NDM-1 producing P. *aeruginosa* isolates from HCB

Isolate ID	Isolation date	Specimen	Susceptibility profile (MIC, mg/L)														ST	WGS resistome	
			TZP (R > 16)	CAZ (R > 8)	FEP (R > 8)	IPM (R > 4)	MEM (R > 8)	AMK (R > 16)	TOB (R > 2)	CIP (R > 0.5)	CST (R > 4)	C/T (R > 4)	CZA (R > 8)	FDC (R > 2)	ATM (R > 16)	A/A		Acquired resistome	Mutational resistome
HCB22-1-0337	14 April 2022	Rectal swab	256	>64	>64	>64	>64	>128	>32	>16	1	>32	>32	1	4	4	773	NDM-1, aadA11^a^, rmtB4, qnrVC1, tet(G)	ΔoprD, mexZ (aa80Δ3), mexT (R40H), gyrA (T83I), parC (S87L), glpT (W413X)
HCB22-1-0406	6 May 2022	Rectal swab	128	>64	>64	>64	>64	>128	>32	>16	1	>32	>32	0.5	8	8	773	NDM-1, aadA11^a^, rmtB4, qnrVC1, tet(G)	ΔoprD, mexZ (aa80Δ3), mexT (R40H), gyrA (T83I), parC (S87L), glpT (W413X), mpl (nt111insC)
HCB22-1-0461	20 May 2022	Urine	128	>64	>64	>64	>64	>128	>32	>16	1	>32	>32	0.5	8	8	773	NDM-1, aadA11^a^, rmtB4, qnrVC1, tet(G)	ΔoprD, mexZ (aa80Δ3), mexT (R40H), gyrA (T83I), parC (S87L), glpT (W413X), mpl (nt111insC)
HCB22-2-0842	10 December 2022	Rectal swab	256	>64	>64	>64	>64	>128	>32	>16	1	>32	>32	1	32	32	773	NDM-1, aadA11^a^, rmtB4, qnrVC1, tet(G)	ΔoprD, nalD (T11P), mexZ (aa80Δ3), mexT (R40H), gyrA (T83I), parC (S87L), glpT (W413X)
HCB23-2-0642	23 June 2023	Rectal swab	256	>64	>64	>64	>64	>128	>32	>16	1	>32	>32	0.5	32	32	773	NDM-1, aadA11^a^, rmtB4, qnrVC1, tet(G)	ΔoprD, nalD (T11P), mexZ (aa80Δ3), mexT (R40H), gyrA (T83I), parC (S87L), glpT (W413X)
HCB23-2-0325	4 November 2023	Rectal swab	>256	>64	>64	>64	>64	>128	>32	>16	1	>32	>32	0.25	32	32	773	NDM-1, aadA11^a^, rmtB4, qnrVC1, tet(G)	ΔoprD, nalD (T11P), mexZ (aa80Δ3), mexT (R40H), gyrA (T83I), parC (S87L), glpT (W413X)
HCB22-3-0898	28 October 2022	Biopsy	128	>64	>64	>64	>64	>128	>32	>16	1	>32	>32	0.5	16	16	773	NDM-1, aadA11^a^, rmtB4, qnrVC1, tet(G)	ΔoprD, nalD (T11P), mexZ (aa80Δ3), mexT (R40H), gyrA (T83I), parC (S87L), glpT (W413X)
HCB23-3-1210	11 August 2023	Rectal swab	128	>64	>64	>64	>64	>128	>32	>16	1	>32	>32	0.5	32	32	773	NDM-1, aadA11^a^, rmtB4, qnrVC1, tet(G)	ΔoprD, nalD (T11P), mexZ (aa80Δ3), mexT (R40H), gyrA (T83I), parC (S87L), glpT (W413X)
HCB24-3-0169	6 February 2024	Rectal swab	256	>64	>64	>64	>64	>128	>32	>16	1	>32	>32	0.25	16	16	773	NDM-1, aadA11^a^, rmtB4, qnrVC1, tet(G)	ΔoprD, nalD (T11P), mexZ (aa80Δ3), mexT (R40H), gyrA (T83I), parC (S87L), glpT (W413X)
HCB24-3-0220	14 February 2024	Rectal swab	128	>64	>64	>64	>64	>128	>32	>16	1	>32	>32	0.5	16	16	773	NDM-1, aadA11^a^, rmtB4, qnrVC1, tet(G)	ΔoprD, nalD (T11P), mexZ (aa80Δ3), mexT (R40H), gyrA (T83I), parC (S87L), glpT (W413X)
HCB23-4-0932	5 September 2023	Rectal swab	256	>64	>64	>64	>64	>128	>32	>16	1	>32	>32	0.5	32	32	773	NDM-1, aadA11^a^, rmtB4, qnrVC1, tet(G)	ΔoprD, nalD (T11P), mexZ (aa80Δ3), mexT (R40H), gyrA (T83I), parC (S87L), glpT (W413X)
HCB23-6-1048	29 September 2023	Rectal swab	256	>64	>64	>64	>64	>128	>32	>16	1	>32	>32	0.5	>64	>64	357	VEB-1, NDM-1, aph(6)-Id, aph(3′′)-Ib, aac(3)-Id, aac(6′)-Il, rmtB4, qnrVC1, msr(E), mph(E), ARR-2, tet(G), dfrB5	oprD (nt1241Δ4), nalD (nt398Δ2), mexB (SV1041EA), ftsI (V537L), gyrA (T83I), parC (S87L), ampDh3 (R134C), parS (A13T), glpT (W275X)
HCB23-5-1031	26 September 2023	Wound swab	256	>64	>64	>64	>64	>128	>32	>16	1	>32	>32	0.5	32	32	773	NDM-1, aadA11^a^, rmtB4, qnrVC1, tet(G)	ΔoprD, nalD (T11P), mexZ (aa80Δ3), mexT (R40H), gyrA (T83I), parC (S87L), glpT (W413X)
HCB23-7-1060	10 June 2023	Urine	>256	>64	>64	>64	>64	>128	>32	>16	1	>32	>32	2	32	32	773	NDM-1, aadA11^a^, rmtB4, qnrVC1, tet(G)	ΔoprD, nalD (T11P), mexZ (aa80Δ3), mexT (R40H), gyrA (T83I), parC (S87L), glpT (W413X)
HCB23-8-1115	23 October 2023	Rectal swab	256	>64	>64	>64	>64	>128	>32	>16	1	>32	>32	1	32	32	773	NDM-1, aadA11^a^, rmtB4, qnrVC1, tet(G)	ΔoprD, nalD (T11P), mexZ (aa80Δ3), mexT (R40H), gyrA (T83I), parC (S87L), glpT (W413X)
HCB24-9-0131	26 January 2024	Rectal swab	256	>64	>64	>64	>64	>128	>32	>16	1	>32	>32	1	32	32	773	NDM-1, aadA11^a^, rmtB4, qnrVC1, tet(G)	ΔoprD, nalD (T11P), mexZ (aa80Δ3), mexT (R40H), gyrA (T83I), parC (S87L), glpT (W413X)
HCB24-10-0229	19 February 2024	Blood culture	256	>64	>64	>64	>64	>128	>32	>16	1	>32	>32	2	32	32	773	NDM-1, aadA11^a^, rmtB4, qnrVC1, tet(G)	ΔoprD, nalD (T11P), mexZ (aa80Δ3), mexT (R40H), gyrA (T83I), parC (S87L), glpT (W413X)

TZP, piperacillin/tazobactam; CAZ, ceftazidime; FEP, cefepime; IPM, imipenem; MEM, meropenem; AMK, amikacin; TOB, tobramycin; CIP, ciprofloxacin; CST, colistin; C/T, ceftolozane/tazobactam; CZA, ceftazidime/avibactam; FDC, cefiderocol; ATM, aztreonam; A/A, aztreonam/avibactam.

^a^aadA11 like (98.6% amino acid identity).

The cgMLST analysis of the ST773 isolates showed that they were closely related (range: 0–13 allele differences), overlapping the intrapatient minimum and maximum allele differences (0–4) with the interpatient differences (0–7).

The first ST773 NDM-1-producing *P. aeruginosa* isolate was detected in a 41-year-old woman, who was admitted to the oncology ward in February 2022. She was originally from Ukraine, where she had undergone breast cancer surgery before being relocated to Spain. Next, an NDM-1-producing *P. aeruginosa* isolate was detected in October 2022 in a 72-year-old woman (Patient 3) who was admitted to the hepatology ward without previous travel history. As these patients did not overlap in time, environmental reservoirs such as high-touch surfaces and sinks were investigated; however, none was identified. Later, 13 more NDM-1-producing *P. aeruginosa* isolates were detected in 8 additional patients admitted to the traumatology, hepatology and oncology wards. The complete spatio-temporal distribution of isolates (*n* = 17) and patient admissions are presented in Figure S1 (available as Supplementary data at *JAC-AMR* Online).

In the ST773-HCB isolates, the *bla*_NDM-1_ gene was chromosomally encoded, along with a bleomycin resistance gene, both located between two IS*91*-family transposases within an ∼117 kb integrative conjugative element (ICE) (Figure S2). Further characterization revealed that this ICE was identical to a *bla*_NDM-1_ ICE recently described in a NDM-1-producing *P. aeruginosa* isolate (NDM-Pa-1, OQ806931.1) obtained from an Ukrainian patient attending a hospital in Madrid (>99.99% identity, 116996/116997 bp).^17^ This ICE was flanked by 23 bp *attL* and *attR* sequences (GTCTCGTTTCCCGCTCCAAACAT) and additionally contained two different aminoglycoside resistance genes (*aadA11*-like and *rmtB4*), a quinolone resistance pentapeptide repeat protein (QnrVC1) and a tetracycline efflux protein (TetG). Likewise, genes involved in the mobilization, integration and maintenance of pathogenicity genomic islands were identified.

HCB-ST773 isolates were resistant to all antibiotics tested except colistin and cefiderocol and displayed variable resistance to aztreonam and aztreonam/avibactam. To completely explain the resistance profiles, we further explored the presence of chromosomal resistance mutations. All ST773 isolates lacked the *oprD* gene due to a 2084 bp deletion (Δnt1043814–1045898, NC_002516.2), which had likely occurred as a result of a homologous recombination between a repetitive sequence of 13 bp (CAAGCTGGGGCGG) located upstream and downstream of the deleted region. Antibiotic resistance mutations were also detected in the genes coding for the glycerol-3-phosphate transporter (*glpT*-W413X), the DNA gyrase subunit A (*gyrA*-T83I) and the DNA topoisomerase IV subunit A (*parC*-S87L), as well as in MexXY (*mexZ*-aa80Δ3) and MexEF-OprN (*mexT-*R40H) efflux pump regulators. Furthermore, with the exception of the first isolate from Patient 1 (HCB22-1-0337), all isolates showed additional mutations either in *mpl* (nt111insC, *n* = 2), which participates in the regulation of the chromosomal cephalosporinase AmpC, or in the MexAB-OprM efflux-pump regulator *nalD* (T11P, *n* = 14) (Table 1). The impact of the *mpl* and *nalD* mutations on the increased expression of *ampC* (25-fold) or *mexB* (4-fold) was confirmed by RT–qPCR in two representative isolates showing each type of mutation (Table 2). As shown in Table 1, the presence of the *nalD* mutation leading to MexAB-OprM overexpression was clearly associated with the acquisition of aztreonam and aztreonam/avibactam resistance. Likewise, the *mexZ* mutation was confirmed to lead to MexXY overexpression but, in contrast, the detected mutation in *mexT* does not drive MexEF-OprN overexpression and is therefore likely a natural polymorphism (Table 2).

**Table 2.** Expression levels of *ampC*, *mexB*, *mexF* and *mexY* of one representative HCB isolate from each unique mutational resistome pattern

Isolate ID	Gene expression (mean ± SD)^a^				Mutations in genes that regulate the expression of AmpC or efflux pumps
	*ampC*	*mexY*	*mexF*	*mexB*	
HCB22-1-0337	0.5 ± 0.1	18.0 ± 2.1	0.2 ± 0.03	0.6 ± 0.1	*mexZ* (aa80Δ3), *mexT* (R40H)
HCB22-1-0461	24.5 ± 3	19.3 ± 0.6	1.0 ± 0.1	1.1 ± 0.0	*mexZ* (aa80Δ3), *mexT* (R40H), *mpl* (nt111insC)
HCB23-7-1060	0.4 ± 0.1	11.9 ± 3.8	0.2 ± 0.0	4.0 ± 1.0	*mexZ* (aa80Δ3), *mexT* (R40H), *nalD* (T11P)

^a^Gene expression levels respect those of the *P. aeruginosa* reference strain PAO1. According to previous works, breakpoints for defining overexpression were established at a 10-fold increase for *ampC*, *mexY* and *mexF*, and 3-fold for *mexB*.^23^

Finally, it should be mentioned that in the ST357 isolate (HCB23-6-1048), the *bla*_NDM-1_ gene was accompanied by a *bla*_VEB-9_ (also designated *bla*_VEB-1a_) ESBL gene, several genes encoding for different aminoglycoside resistance determinants (*aph(6)-Id*, *aph(3′′)-Ib*, *aac(3)-Id*, *aac(6′)-Il*, *rmtB4*), a quinolone resistance pentapeptide repeat protein (*qnrVC1*) and other acquired antibiotic resistance genes (Table 1). The presence of all these acquired resistance determinants correlated well with the antibiotic susceptibility profile, as this isolate was resistant to all antibiotics except colistin and cefiderocol. Recently, Rossell *et al.*^29^ investigated the origin of carbapenemase-producing *P. aeruginosa* ST357 in the Netherlands, comparing the resistome of their isolates with international ones, and concluded that this clone was introduced to the Netherlands via repatriation of critically ill patients from Kenya. The acquired resistome of our isolate (HCB23-6-1048) was identical to an Indian subclade (SNP cluster PDS000019917) of the international collection investigated in that work. Interestingly, HC23-6-1048 was isolated in a 69-year-old man admitted to the traumatology ward who had been previously admitted to an ICU in a hospital in Jaipur (India) with sepsis. We then compared the genetic environment of the *bla*_NDM-1_ in our isolate with a Dutch representative isolate of the Kenyan subclade (isolate RIVM_C050529); we found that while in the Dutch isolate the *bla*_NDM-1_ gene was located within a resistance island containing other resistance determinants such as *bla*_VEB-9_, in our isolate the *bla*_NDM-1_ gene was alone (Figure S2), the *bla*_VEB-9_ gene being located distantly in the chromosome. Thus, both subclades had apparently acquired the *bla*_NDM-1_ and *bla*_VEB-9_ genes through different events, highlighting the ability of this high-risk clone to acquired foreign resistance determinants.

### Global analysis of the genomic epidemiology and resistome of ST773 *P. aeruginosa*

**Figure 1.**
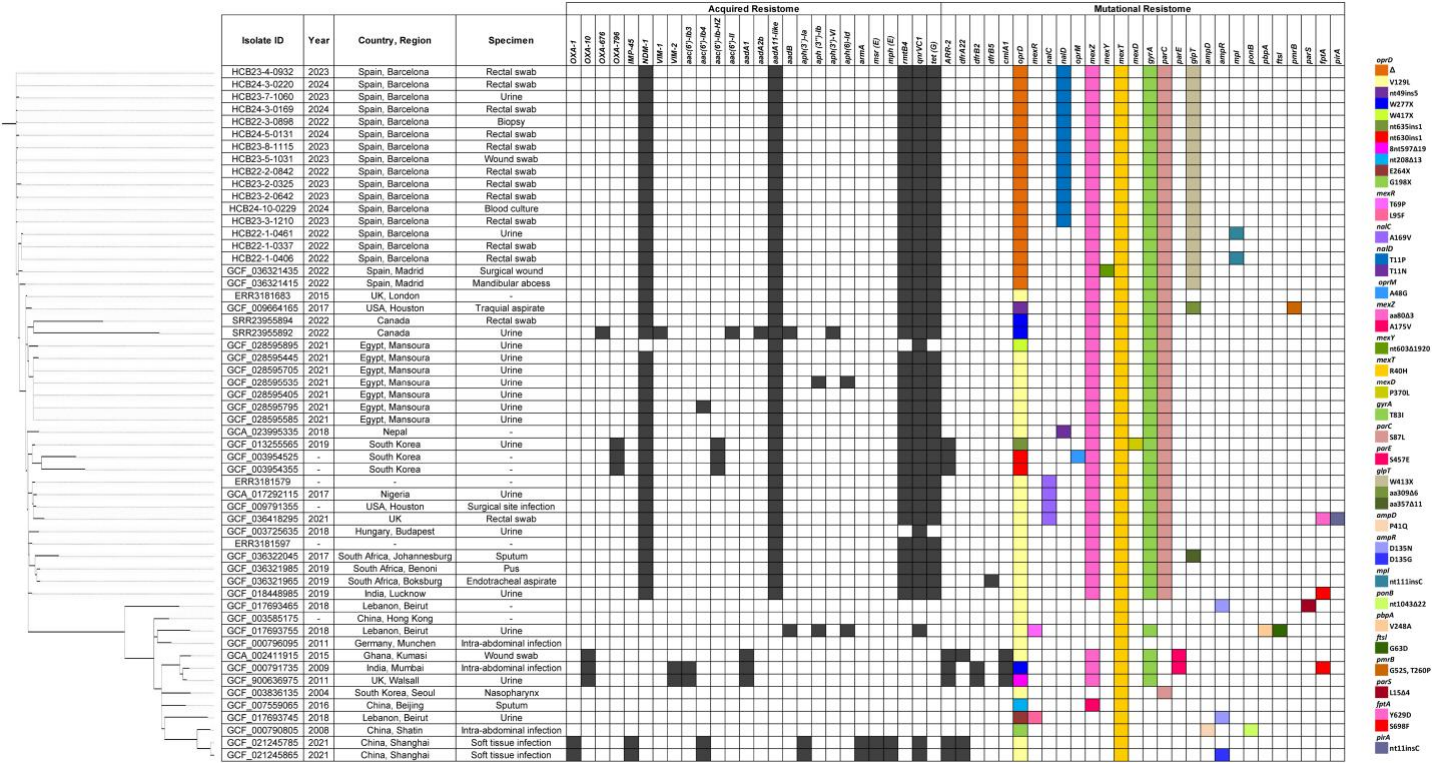
Core-genome SNP-based phylogenetic reconstruction of ST773 *P. aeruginosa* isolates. In the acquired resistome, presence/absence is represented by black/white, respectively, whereas for the mutational resistome the colour code is represented on the right-hand side (each colour of each column corresponds to a single mutation). OXA-676 and OXA-796 β-lactamases are OXA-10 and OXA-1 derivatives, respectively.

To infer the potential geographical origin of the NDM-1-producing ST773 HCB *P. aeruginosa* isolates, 40 additional ST773 *P. aeruginosa* genomes obtained between 2004 and 2022 from patients in different countries worldwide were downloaded from public databases for phylogenetic and resistome comparisons. This international ST773 collection included 26 NDM-1 producers but also included 14 isolates that did not harbour a *bla*_NDM-1_ gene, some of them containing a different carbapenemase-encoding gene such as *bla*_IMP-45_ or *bla*_VIM-2_ (Figure 1).

**Figure 2.**
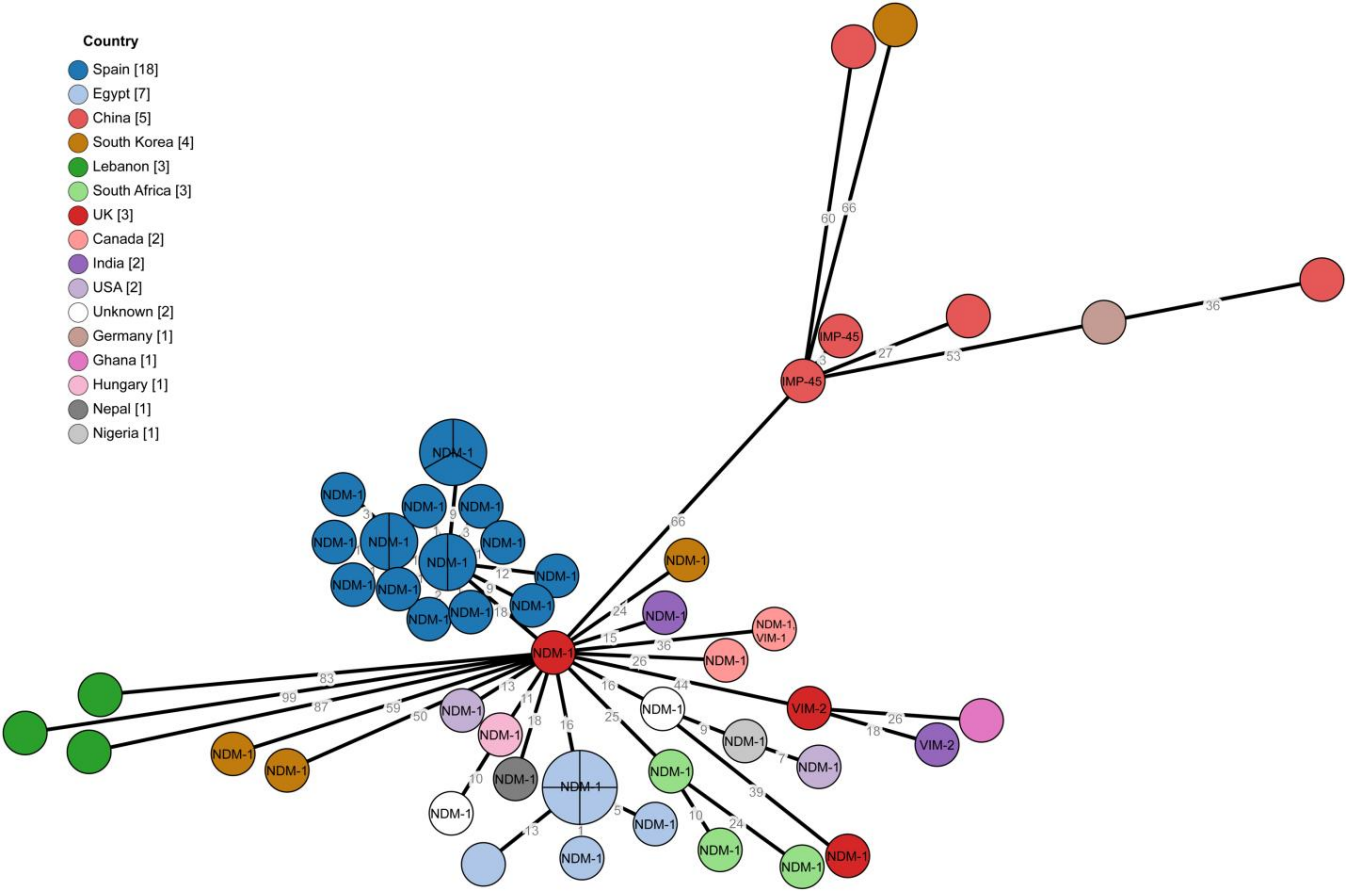
Core-genome MLST analysis of the ST773 *P. aeruginosa* global collection. Each colour represents the country of origin of the isolates.

The genetic relatedness of the HCB isolates and the international ST773 *P. aeruginosa* genomes was explored, combining both SNP (Figure 1) and gene-by-gene approaches (Figure 2). As shown in Figure 1, NDM-1 producers were demonstrated to be closely related and clustered together, forming a distinct branch separated from non-NDM-1 producers. By cgMLST analysis, a median allele difference within the entire collection of 47 (range 0–143) was determined to be higher among the non-NDM-1 isolates compared with the NDM-1 ones (73 versus 29) (Figure 2). Allele differences confirmed that the HCB isolates were closely related to one another and also to the two isolates included in the international collection from Madrid (range: 0–16). Notably, the isolates from Patient 1 (HCB-1) and the two isolates from Madrid were the most genetically distant, allele differences ranging from 9 to 16 compared with the 0–7 allele differences documented among the other HCB isolates. Moreover, we found that among these patients, intrapatient minimum and maximum allele differences (0–4) overlapped with the interpatient range (0–7). Altogether, these findings pointed to a common geographical origin for the introduction of this NDM-1 ST773 clone in both Spanish hospitals (Ukraine) followed by sustained local interpatient transmission events after its introduction into the HCB.

As shown, with the single exception of the Hungarian isolate (GCF_003725635), all NDM-1 producers had the same acquired resistance determinants as those detected in the HCB isolates [*aadA11-*like, *rmtB4*, *qnrVC1* and *tet*(G)], as well as the antibiotic resistance mutations found in *mexZ*, *mexT*, *gyrA* and *parC* chromosomal genes. However, other HCB genetic markers such as the inactivating mutation in *glpT* (W413X) and the OprD deletion, were only found in the two NDM-1-producing isolates obtained from two Ukrainian patients attending a hospital in Madrid, genomic features that along with epidemiological data suggest a common origin of these isolates. Moreover, the characterization of the *bla*_NDM-1_ genetic environment in isolate HCB22-1-0461 (Figure S2) additionally supports this hypothesis, as it was shown to be located in the same 117 kb ICE (>99.99% identity, 116996/116997) and inserted within exactly the same chromosomal position as isolate NDM-Pa-1 from Madrid (OQ806931.1).^16^

On the other hand, non-NDM-1 producers were less clonal and exhibited more variable resistomes (Figure 1). Among them, just the mutation in *mexT* was conserved, suggesting that the acquisition of this mutation occurred a long time ago and before the acquisition of other antibiotic resistance genes/mutations.

## Conclusions

We report the concomitant detection of NDM-1-producing *P. aeruginosa* isolates belonging to two distinct high-risk clones, ST357 and ST773, in a tertiary Spanish hospital. Using a combination of molecular and WGS approaches, we have characterized their resistomes in depth, compared them with those of international collections and determined by phylogenetic analysis their most likely geographical origins and routes of transmission.

NDM-1-producing ST773 *P. aeruginosa* isolates detected worldwide showed a higher degree of similarity. Genomic features and phylogenetic analysis of the HCB ST773 isolates strongly linked them with those recently reported in two Ukrainian patients from a hospital in Madrid, isolates that have been linked to NDM-1-producing ST773 strains found in Ukrainian patients in the Netherlands.^17^ Thus, our findings provide further evidence that the ongoing war in Ukraine has been a key factor driving the clonal expansion of this NDM-1-ST773 *P. aeruginosa* strain across Europe. In addition, we confirmed sustained local interpatient transmission events following the initial introduction of this clone into a tertiary hospital in Barcelona. Finally, our detailed resistome analysis identified common elements to all NDM-1 ST773 isolates and specific features of the lineages disseminated in Europe linked to the Ukrainian war, as well as genomic markers of resistance evolution during its intrahospital dissemination.

In summary, this work evidences the transborder spread and intrahospital dissemination and evolution of the emerging ST773-NDM-1 *P. aeruginosa* high-risk clone. Likewise, this work underscores the importance of surveillance to prevent the spread of successful high-risk clones and antibiotic resistance determinants worldwide, positing genomic epidemiology and resistome analysis as essential tools to understand their behaviour and transmission patterns.

## Supplementary Material

dlaf063_Supplementary_Data

## Data Availability

Read files for isolates sequenced in this study have been deposited in the European Nucleotide Archive under project number PRJEB75515.

## Supplementary data

Figures S1 and S2 are available as Supplementary data at *JAC-AMR* Online.
