# Supplementary material for: Analysis of intrahospital and global dissemination and resistome dynamics of NDM-1-producing ST773 Pseudomonas aeruginosa high-risk clone
Source: JAC Antimicrob Resist. 2025 Apr 22;7(2):dlaf063. doi: 10.1093/jacamr/dlaf063 (PMC12013283; doi:10.1093/jacamr/dlaf063)
Supplement: dlaf063_Supplementary_Data [file dlaf063_supplementary_data.docx]

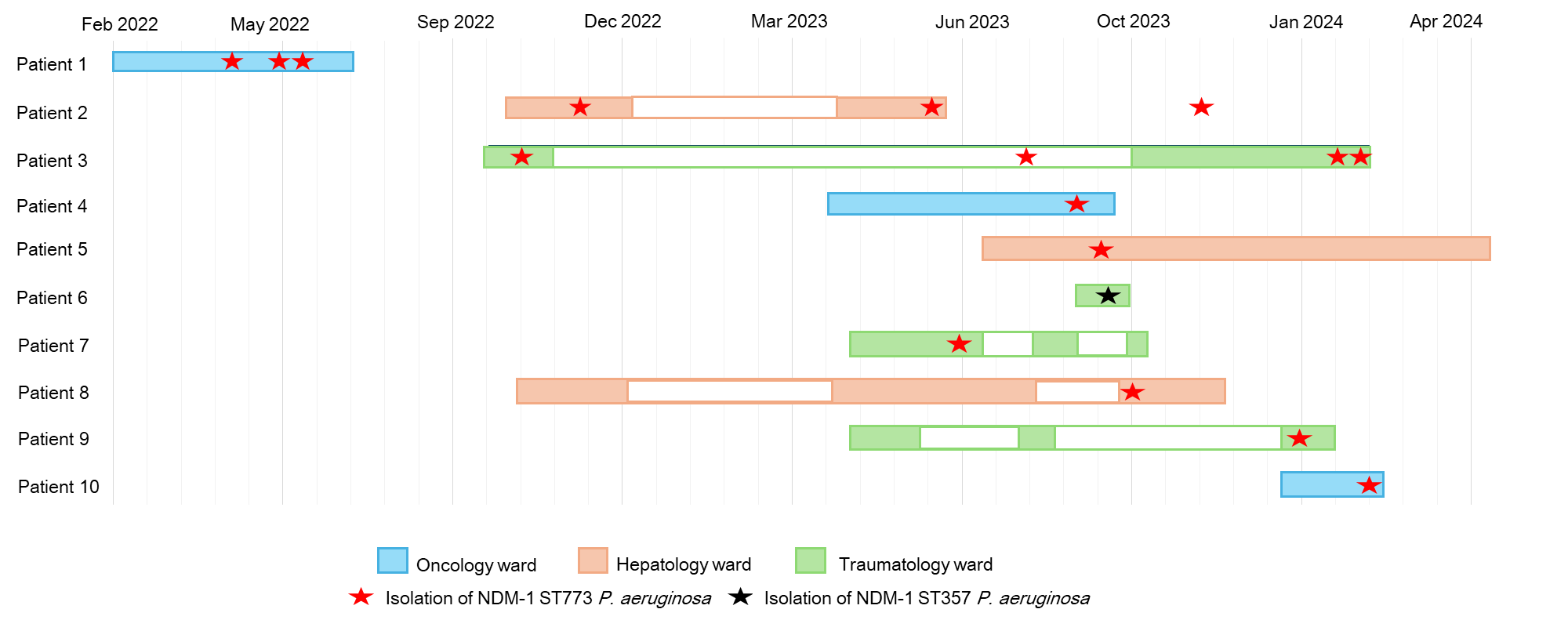
**Figure S1. Spatio-temporal distribution of *Pseudomonas aeruginosa* NDM-1-producing isolates and admissions of patients at the Hospital Clinic of Barcelona.** Coloured boxes represent elapsed time between hospital admission and discharge, representing white boxes periods between admissions.


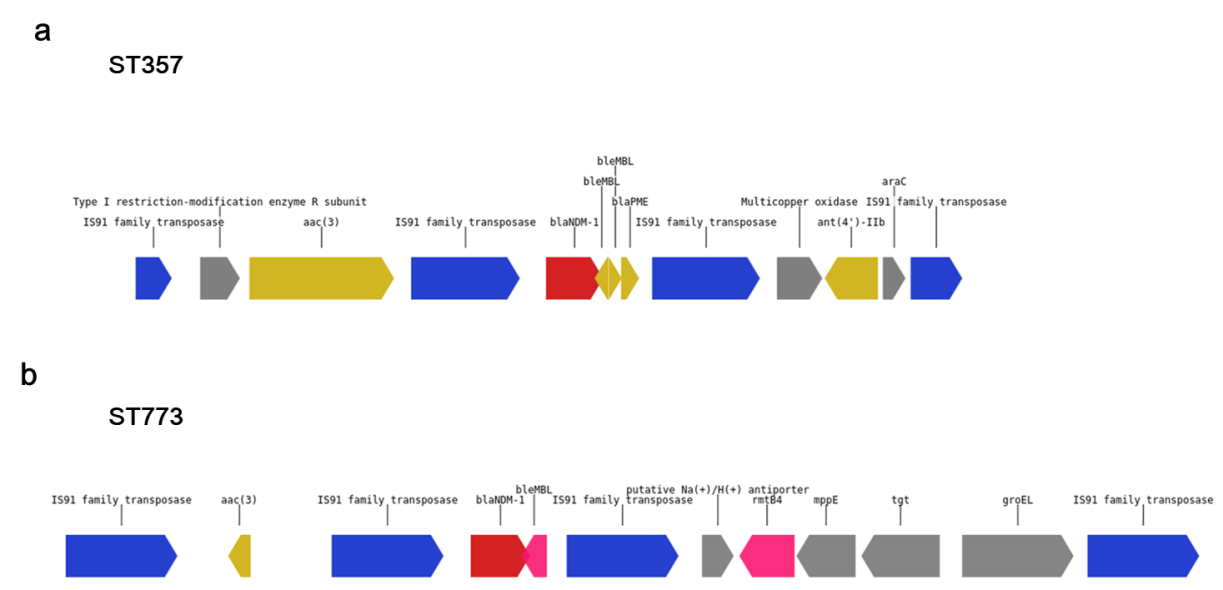


**Figure S2. Schematic representation of the genetic environments of the *bla*_NDM-1_ gene in (a) ST357 (HCB23-6-1048) and (b) ST773 (HCB22-1-0337, HCB22-1-0461 and HCB23-7-1060) representative *P. aeruginosa* isolates.** The *bla*_NDM-1_ gene has been indicated in red. The pink colour indicates other complete antibiotic resistance genes detected while the yellow indicates either partial genes or complete genes with an identity <90% compared to the reference (*ant(4’)-IIb*).
